# TIFA Signaling in Gastric Epithelial Cells Initiates the *cag* Type 4 Secretion System-Dependent Innate Immune Response to *Helicobacter pylori* Infection

**DOI:** 10.1128/mBio.01168-17

**Published:** 2017-08-15

**Authors:** Alevtina Gall, Ryan G. Gaudet, Scott D. Gray-Owen, Nina R. Salama

**Affiliations:** a Molecular and Cellular Biology Graduate Program, University of Washington, Seattle, Washington, USA; b Human Biology Division, Fred Hutchinson Cancer Research Center, Seattle, Washington, USA; c Department of Molecular Genetics, University of Toronto, Toronto, Ontario, Canada; d Howard Hughes Medical Institute and Departments of Microbial Pathogenesis and of Immunobiology, Yale University, New Haven, Connecticut, USA; e Department of Microbiology, University of Washington School of Medicine, Seattle, Washington, USA; New York University

**Keywords:** CagA, *Helicobacter pylori*, NOD1, TIFA

## Abstract

*Helicobacter pylori* is a bacterial pathogen that colonizes the human stomach, causing inflammation which, in some cases, leads to gastric ulcers and cancer. The clinical outcome of infection depends on a complex interplay of bacterial, host genetic, and environmental factors. Although *H. pylori* is recognized by both the innate and adaptive immune systems, this rarely results in bacterial clearance. Gastric epithelial cells are the first line of defense against *H. pylori* and alert the immune system to bacterial presence. Cytosolic delivery of proinflammatory bacterial factors through the *cag* type 4 secretion system (*cag*-T4SS) has long been appreciated as the major mechanism by which gastric epithelial cells detect *H. pylori*. Classically attributed to the peptidoglycan sensor NOD1, recent work has highlighted the role of NOD1-independent pathways in detecting *H. pylori*; however, the bacterial and host factors involved have remained unknown. Here, we show that bacterially derived heptose-1,7-bisphosphate (HBP), a metabolic precursor in lipopolysaccharide (LPS) biosynthesis, is delivered to the host cytosol through the *cag*-T4SS, where it activates the host tumor necrosis factor receptor-associated factor (TRAF)-interacting protein with forkhead-associated domain (TIFA)-dependent cytosolic surveillance pathway. This response, which is independent of NOD1, drives robust NF-κB-dependent inflammation within hours of infection and precedes NOD1 activation. We also found that the CagA toxin contributes to the NF-κB-driven response subsequent to TIFA and NOD1 activation. Taken together, our results indicate that the sequential activation of TIFA, NOD1, and CagA delivery drives the initial inflammatory response in gastric epithelial cells, orchestrating the subsequent recruitment of immune cells and leading to chronic gastritis.

## IMPORTANCE

*H. pylori* is a globally prevalent cause of gastric and duodenal ulcers and cancer. *H. pylori* antibiotic resistance is rapidly increasing, and a vaccine remains elusive. The earliest immune response to *H. pylori* is initiated by gastric epithelial cells and sets the stage for the subsequent immunopathogenesis. This study revealed that host TIFA and *H. pylori*-derived HBP are critical effectors of innate immune signaling that account for much of the inflammatory response to *H. pylori* in gastric epithelial cells. HBP is delivered to the host cell via the *cag*-T4SS at a time point that precedes activation of the previously described NOD1 and CagA inflammatory pathways. Manipulation of the TIFA-driven immune response in the host and/or targeting of ADP-heptose biosynthesis enzymes in *H. pylori* may therefore provide novel strategies that may be therapeutically harnessed to achieve bacterial clearance.

## INTRODUCTION

*Helicobacter pylori* is a Gram-negative bacterium that colonizes greater than 50% of the world’s population. The only known niche of *H. pylori* is the human stomach, where the bacterium resides in intimate contact with gastric epithelial cells, causing inflammation that, in a subset of infected individuals, progresses to gastric and duodenal ulcers and cancer (1). It has been estimated that *H. pylori* has resided with its human host for at least 100,000 years, predating modern human migration out of Africa, making it the oldest known and one of the most successful human pathogens (2, 3).

*H. pylori* is able to achieve chronic colonization due in part to its ability to evade host immune responses. A number of *H. pylori* pathogen-associated molecular patterns (PAMPs) avoid stimulation of their cognate pattern recognition receptors (PRRs). The lipopolysaccharide (LPS) of *H. pylori* is tetra-acetylated, making it a poor ligand for Toll-like receptor 4 (TLR4) (4), and divergent flagellin monomer sequences abrogate its interaction with TLR5 (5, 6). The O-antigen of *H. pylori* LPS contains Lewis antigens which mimic host receptors and facilitate immune escape (7). Furthermore, the unique dephosphorylated lipid A structure in *H. pylori* confers resistance to host antimicrobial peptides, as well as to the antibiotic polymyxin B (8). Despite sophisticated evasion strategies, several virulence factors induce a robust immune response. *H. pylori*-mediated gastritis is initiated by proinflammatory cytokines released by gastric epithelial cells, as well as neutrophils and macrophages that are recruited to the site of infection (9). Cytotoxin-associated gene A (CagA) has been extensively characterized as a major contributor to epithelial cell transformation that can result in gastric cancer. Infection with *cagA*-positive (*cagA*^+^) strains is associated with increased inflammation and subsequent development of peptic ulcers and gastric adenocarcinoma (10, 11). CagA is encoded within a region of the chromosome known as the *cag* pathogenicity island (*cag*-PAI). Along with *cagA*, *cag*-PAI contains genes required for assembly of a type IV secretion system (T4SS). CagA is injected directly into epithelial cells via the *cag*-T4SS, which acts as a molecular syringe. Once CagA is delivered into the host cytosol, it is phosphorylated by host kinases, leading to aberrant host cell signaling. After several months of experimental infection performed with a single *H. pylori* strain, mice and rhesus macaques accumulated bacterial strain variants that contained mutations in the *cagY* gene which rendered the *cag*-T4SS nonfunctional. Interestingly, strains with a functional *cag*-T4SS were also recovered from the same animals (12, 13), suggesting that the ability to switch the *cag*-T4SS on or off is advantageous for the bacteria.

Along with CagA, the *cag*-T4SS delivers bacterial factors that are known to activate the global transcription factor NF-κB, leading to the upregulation of proinflammatory genes and production of cytokines such as interleukin-8 (IL-8) (14). IL-8 is a major chemokine for neutrophil recruitment to the site of infection and has been extensively used as a physiologically relevant readout of the NF-κB inflammatory pathway. Active *H. pylori* infection is characterized by extensive neutrophil infiltration of the gastric mucosa, where these cells not only recruit lymphocytes, which establish chronic inflammation, but also contribute to tissue damage through production of reactive oxygen species (15). Prior to this study, most of the *cag-*T4SS-dependent IL-8 response in gastric epithelial cells had been attributed to NOD1 activation on the basis of a foundational study demonstrating that NOD1 activation by *H. pylori* peptidoglycan (PG) leads to NF-κB activation and IL-8 production and that *Nod1*-deficient mice are more susceptible to *H. pylori* infection (16). Although several groups have challenged the notion that NOD1-dependent responses predominate in *H. pylori*-infected gastric epithelial cells (17–19), comprehensive understanding of the relevant pathogen recognition pathways has remained elusive. Interestingly, recent work has suggested that additional bacterial products may be delivered by the *cag*-T4SS. TLR9-dependent inhibition of IL-8 induction in reporter cell lines and suppression of the *H. pylori* load in *Tlr9*^−/−^ mice suggest that bacterial DNA containing inhibitory CpG motifs can also transit the *cag*-T4SS, though in this case with the result of dampening NF-κB signaling (20, 21).

In this study, we identified tumor necrosis factor receptor-associated factor (TRAF)-interacting protein with forkhead-associated domain (TIFA) as a critical innate signaling component downstream of *cag*-T4SS-induced inflammation. TIFA has been extensively studied in the context of its interaction with TRAF6 and ability to activate NF-κB (22–24). Recent work has revealed that TIFA is specifically activated by the cytosolic presence of the bacterial metabolite heptose-1,7-bisphosphate (HBP), a metabolic intermediate in LPS biosynthesis (25, 26). HBP is highly conserved among Gram-negative bacteria but is absent from eukaryotic cells. Thus, TIFA-dependent detection of HBP represents a novel innate immune-sensing pathway that is distinct from classical TLR or NOD family pattern recognition receptor-driven signaling cascades. HBP is detected in the host cytosol and has been proposed to gain access to this compartment through dynamin-dependent endocytosis of extracellularly released HBP (*Neisseria*, *Salmonella enterica* serovar Typhimurium) and phagolysosomal degradation of engulfed bacteria within macrophages (*Escherichia coli*) as well as through intracellular bacterial replication (*Shigella flexneri*) (25–28). Here, we reveal an additional mechanism whereby HBP is presented to the TIFA signaling pathway: type IV secretion system translocation. In the context of *H. pylori* infection, HBP is delivered to the host cytoplasm through the *cag*-T4SS, where it activates an acute TIFA-mediated innate immune response in gastric epithelial cells. Furthermore, we show that host NOD1 and the bacterial CagA toxin are sequentially responsible for the *H. pylori*-induced inflammation that follows the early TIFA response.

## RESULTS

### *NOD1* gene targeting does not eliminate the *cag-*T4SS-dependent IL-8 response in gastric epithelial cells.

**FIG 1:**
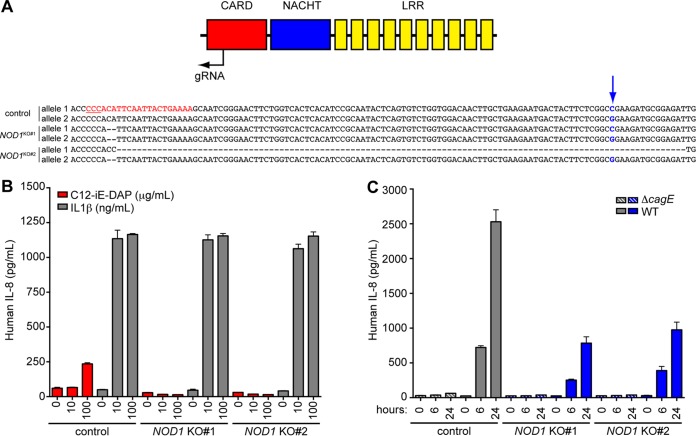
NOD1 contributes only partially to *H. pylori* detection. (A) Schematic representation of NOD1 structural domains and location of the guide RNA used to generate CRISPR-targeted, clonally selected *NOD1* KO cell lines. A portion of the sequence of each allele in control targeted cells is shown with the guide RNA sequence highlighted in red and the protospacer adjacent motif (PAM) underlined. The SNP used to distinguish allele 1 from allele 2 is shown in blue with an arrow. The deletions generated in the *NOD1* KO#1 and KO#2 cell lines are represented by dashes. (B) IL-8 ELISA performed on control targeted *NOD1* KO#1 and KO#2 AGS cells treated with IL-1β or C12-iE-DAP. Each condition was tested in triplicate, with bars showing means and standard deviations. (C) Control or *NOD1* targeted cells were cocultured with wild-type (WT) G27 *H. pylori* or the Δ*cagE* isogenic mutant at a multiplicity of infection (MOI) of 10, and IL-8 concentrations in the supernatant were measured by ELISA. Each condition was tested in triplicate, with bars showing means and standard deviations. Data shown in panels B and C are representative of at least three independent experiments.

To determine whether NOD1 was solely responsible for the IL-8 response in gastric epithelial cells, we used clustered regularly interspaced short palindromic repeat (CRISPR)/Cas9 gene editing tools to target *NOD1* in a human gastric epithelial cell line (AGS cells). We designed a guide RNA targeting the N-terminal caspase recruitment domain (CARD) of NOD1, which is required for interaction with its downstream signaling partner RIP2 (29). We clonally selected two *NOD1* knockout (KO) cell lines and characterized the deletions on both alleles of *NOD1*. The *NOD1* KO#1 cell line contained identical 2-bp deletions adjacent to the PAM motif, while the *NOD1* KO#2 cell line contained a 112-bp deletion on the first allele and a 2-bp deletion on the second allele (Fig. 1A). To assess the functional loss of NOD1, we treated control or *NOD1*-targeted cells with a NOD1-specific ligand (C12-iE-DAP). We found that the response to NOD1-specific ligand was completely abrogated in *NOD1* KO cells compared to control targeted cells, and yet all cell lines retained the IL-8 response to IL-1β, a NOD1-independent stimulus (Fig. 1B). When we cocultured control-targeted AGS cells with G27, a wild-type clinical isolate of *H. pylori*, we found that the cells produced IL-8 in response to infection and that, consistent with published data, the IL-8 response was completely dependent on the presence of an intact *cag*-T4SS. Surprisingly, *NOD1* KO attenuated but did not abolish IL-8 production in response to infection with wild-type *H. pylori* (Fig. 1C), suggesting that an additional host pathway(s) is activated by bacterial factors delivered through the *cag*-T4SS.

### *TIFA* gene targeting in epithelial cells suppresses the IL-8 response to *H. pylori* infection.

**FIG 2:**
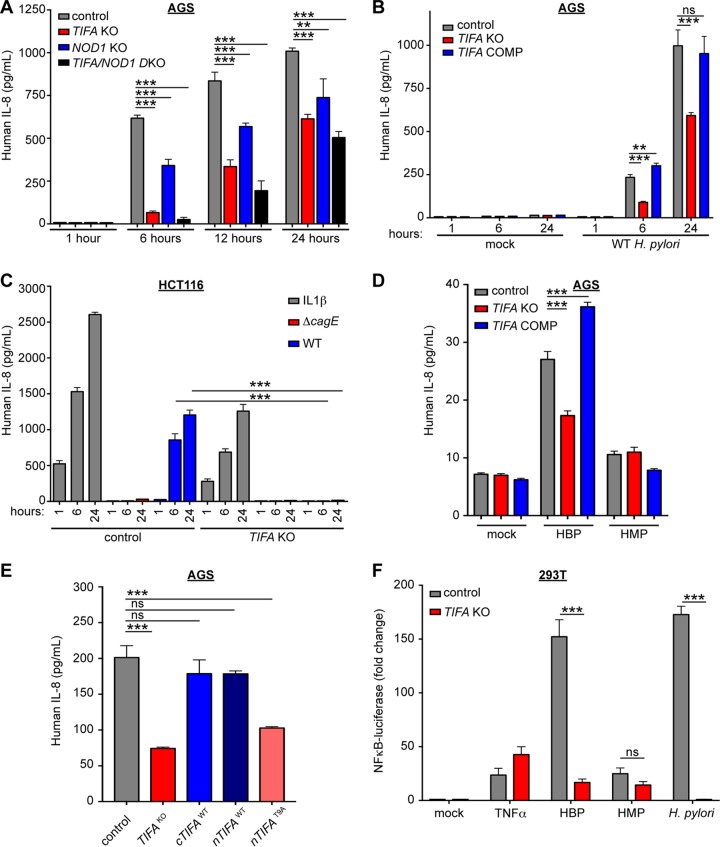
TIFA is required for the early *cag-*T4SS-dependent NF-κB-driven immune response in gastric epithelial cells. (A) Control, *TIFA*-targeted *NOD1* KO#1, or *TIFA*-targeted *NOD1* KO#1 AGS cells (DKO) were cocultured with wild-type G27 *H. pylori*, and IL-8 concentrations in the supernatant were measured by ELISA. (B) Control AGS cells, *TIFA*-targeted AGS cells, or *TIFA*-targeted AGS cells stably complemented (COMP) with full-length TIFA were mock treated or cocultured with wild-type *H. pylori*, and IL-8 concentrations in the supernatant were measured by ELISA. (C) Control or *TIFA*-targeted colorectal adenocarcinoma cells (HCT116) were treated with IL-1β and wild-type or Δ*cagE* isogenic mutant *H. pylori*, and IL-8 concentrations were measured in the supernatant. (D) Control or *TIFA* KO AGS cells were reversibly permeabilized with digitonin for 15 min and mock treated or stimulated with HBP (*ΔgmhB*) or HMP (*ΔhldA*) containing *Neisseria* lysates. Supernatants were collected after 6 h, and IL-8 levels were measured by ELISA. (E) Control, *TIFA* KO, C-terminally tagged wild-type TIFA-complemented, N-terminally tagged wild-type, or T9A TIFA-complemented AGS cells were cocultured with *H. pylori*, and IL-8 levels were measured in culture supernatants after 6 h of infection. For coculture experiments (A to C and E), *H. pylori* was added at MOI = 10, supernatant was collected at the indicated time points, and each condition was tested in triplicate, with bars showing means and standard deviations. (F) NF-κB–luciferase activity in wild-type or *TIFA*-targeted 293T cells treated with TNF-α (5 ng/ml), *N. meningitides*-derived HBP and HMP, or wild-type *H. pylori* lysate. Data were normalized to signal from cotransfected *Renilla* luciferase plasmid and are represented as normalized fold changes from mock-treated samples. Each condition was tested in triplicate, with bars showing means with standard deviations. Data shown in panels A to F are representative of results from at least two independent experiments. Statistical significance was determined using ANOVA with Bonferroni correction for multiple comparisons. ns, not significant (*P* > 0.05); **, *P* < 0.01; ***, *P* < 0.001.

Given the recent discovery of TIFA as a critical innate immune signaling component in response to Gram-negative bacteria, we hypothesized that TIFA may be playing a role in *H. pylori* detection by gastric epithelial cells. Using CRISPR, we targeted TIFA in wild-type or *NOD1* KO AGS cells and assessed IL-8 responses following exposure to strain G27, a clinical isolate of *H. pylori*. We determined that in response to *H. pylori* infection, IL-8 responses were significantly attenuated in *TIFA* KO cells, particularly at the early time point (6 h), compared to the control targeted cells (Fig. 2A). To address the reproducibility of NOD1- and TIFA-dependent IL-8 signaling in AGS cells infected with *H. pylori*, we analyzed our data in aggregate across three independent experiments. We found that *H. pylori* induced NOD1-dependent IL-8 production was attenuated at 24 h. However, IL-8 levels at 6 and 12 h in *NOD1* KO cells differed across experiments and, analyzed across biological replicates, were not statistically significantly different from the levels seen with control AGS cells (see Fig. S1A in the supplemental material). *TIFA* KO AGS cells infected with *H. pylori* consistently showed significantly reduced IL-8 levels at 6, 12, and 24 h, both in analysis of a representative experiment (Fig. 2A) and across three independent experiments (Fig. S1A). Because we observed some differences in analyses of representative experiments versus combined data from three independent experiments, we include statistical analysis of biological replicates (Fig. S1). To assess whether TIFA-dependent IL-8 induction was unique to the G27 strain, we cocultured *TIFA* KO or *TIFA NOD1* DKO AGS cells with different *H. pylori* strains (G27, PMSS1, J99, and 26695). We found that, although the overall IL-8 levels differed across strains, all of the strains tested induced TIFA-dependent IL-8 production in AGS cells (Fig. S2A). In addition, we found that the effects of targeting both *NOD1* and *TIFA* were additive, since the *NOD1 TIFA* DKO cells showed a more dramatic decrease in IL-8 responses than was seen in targeting either *NOD1* or *TIFA* alone (Fig. 2A; Fig. S1A; Fig. S2A). This indicates that these two pathogen recognition pathways contribute to *H. pylori* detection and yet are functionally independent. Interestingly, we observed residual IL-8 signal at the later time points, suggesting that in addition to peptidoglycan and HBP, at least one other bacterially derived factor delivered through the *cag*-T4SS is driving an immune response in gastric epithelial cells. To ensure that the effect on IL-8 production was TIFA-dependent and did not result from CRISPR off-target effects, we transduced CRISPR-targeted AGS cells with lentivirus containing the complete TIFA coding sequence and showed that the IL-8 produced in response to *H. pylori* infection in TIFA-complemented cells was restored. We found that at the 6-h time point, the level of IL-8 induced in TIFA-complemented cells was statistically significantly higher than in control targeted cells (*P* = 0.001), which may have been due to *TIFA* overexpression since *TIFA* expression in the complemented cells is driven by the lentiviral MND promoter (Fig. 2B, Fig. S1B). Furthermore, since TIFA signaling relies on oligomerization, which can be caused by protein overexpression, we confirmed that no IL-8 was produced in mock-treated TIFA-complemented cells (Fig. 2B).

10.1128/mBio.01168-17.1 FIG S1  TIFA is required for the early *cag-*T4SS-dependent NF-κB-driven immune response in gastric epithelial cells. (A) Control, *TIFA*-targeted *NOD1* KO#1, or *TIFA*-targeted *NOD1* KO#1 AGS cells (DKO) were cocultured with wild-type *H. pylori*, and IL-8 concentrations in the supernatant were measured by ELISA. (B) Control AGS cells, *TIFA*-targeted AGS cells, or *TIFA*-targeted AGS cells stably complemented with full-length TIFA were cocultured with wild-type *H. pylori*, and IL-8 concentrations in the supernatant were measured by ELISA. (C) Control or *TIFA*-targeted colorectal adenocarcinoma cells (HCT116) were treated with IL-1β and wild-type or *ΔcagE* isogenic mutant *H. pylori*, and IL-8 concentrations were measured in the supernatant. Data in panels A to C were combined from three independent experiments and represent fold changes in IL-8 concentrations relative to control targeted AGS (A and B) or HCT116 (C) cells. (D) Control or *TIFA* KO AGS cells were reversibly permeabilized with digitonin for 15 min and mock treated or stimulated with HBP (Δ*gmhB*) or HMP (*ΔhldA*) containing *Neisseria* lysates. Supernatants were collected after 6 h, and IL-8 levels were measured by ELISA. Data present results from two independent experiments and are represented as fold changes in IL-8 concentrations relative to control targeted AGS cells. (E) Control, *TIFA* KO, C-terminally tagged wild-type TIFA, and N-terminally tagged wild-type or T9A TIFA-complemented AGS cells were cocultured with *H. pylori*, and IL-8 levels were measured in culture supernatants after 6 h of infection. Data present results from two independent experiments and are represented as fold changes in IL-8 concentrations relative to control targeted AGS cells. (F) NF-κB luciferase activity in wild-type or *TIFA*-targeted 293T cells treated with TNF-α (5 ng/ml), *N. meningitides*-derived HBP and HMP, or wild-type *H. pylori* lysate. Data present results from three independent experiments in which NF-κB luciferase signal was normalized to signal from cotransfected *Renilla* luciferase plasmid and are represented as normalized fold changes from mock-treated samples. In A-F, the bars indicate the mean with standard deviation of data from 2 to 3 independent experiments. Statistical significance was determined using ANOVA with Bonferroni correction for multiple comparisons. ns, not significant (*P* > 0.05); *, *P* < 0.05; **, *P* < 0.01; ***, *P* < 0.001. Download FIG S1, TIF file, 0.7 MB. Copyright © 2017 Gall et al. 2017 Gall et al. This content is distributed under the terms of the Creative Commons Attribution 4.0 International license.

10.1128/mBio.01168-17.2 FIG S2  Multiple *H. pylori* strains induce TIFA-dependent signaling in epithelial cells. (A) Control, *TIFA*-targeted, or *TIFA*-targeted *NOD1* KO#1 AGS cells (DKO) were cocultured with the indicated *H. pylori* strains (MOI = 10), and IL-8 concentration in the supernatant were measured by ELISA at 6 and 24 h. (B) NF-κB luciferase activity in wild-type or *TIFA*-targeted 293T cells mock treated or stimulated with *N. meningitides*-derived HBP and HMP or bacterial lysates from the indicated *H. pylori* strains (lysate normalized using OD_600_ measurements). NF-κB luciferase signal was normalized to signal from cotransfected *Renilla* luciferase plasmid, and data are represented as normalized fold changes from mock-treated samples. (A and B) Data are representative of results from two independent experiments in which each strain was tested in triplicate, with bars showing means and standard deviations. Download FIG S2, TIF file, 0.3 MB. Copyright © 2017 Gall et al. 2017 Gall et al. This content is distributed under the terms of the Creative Commons Attribution 4.0 International license.

### Activation of the TIFA-mediated inflammatory response to *H. pylori* is *cag*-T4SS dependent.

The IL-8 response to *H. pylori* in AGS cells is dependent on a functional *cag*-T4SS, since infection with a *cag*-T4SS mutant (strain Δ*cagE*) resulted in complete loss of IL-8 production (Fig. 1C). Similarly to wild-type AGS cells, *TIFA* KO AGS cells do not produce IL-8 in response to Δ*cagE* mutant *H. pylori* (data not shown). Importantly, our data indicate that, without a functional *cag*-T4SS, the TIFA-dependent immune response to *H. pylori* is completely abrogated. To confirm that *H. pylori* activates a TIFA-mediated inflammatory response and that it is dependent on the *cag*-T4SS, we cocultured *H. pylori* with HCT116 cells, a colorectal adenocarcinoma cell line that, like AGS cells, expresses the α5β1 integrin required for *cag*-T4SS attachment and CagA translocation (30, 31) and has a functional TIFA signaling pathway (28). When we infected control or TIFA-targeted HCT116 cells with *H. pylori*, we found that in the absence of TIFA, the IL-8 response was completely abrogated, while the IL-8 response to a TIFA-independent ligand, IL-1β, was intact in both control and *TIFA*-targeted cells. Consistent with our data determined in AGS cells, we found that IL-8 produced by colon epithelial cells in response to *H. pylori* is completely *cag*-T4SS dependent, since no IL-8 was produced when we performed a coculture with a Δ*cagE* mutant (Fig. 2C; Fig. S1C). However, in contrast to AGS cells, the IL-8 induced following *H. pylori* detection in the context of this colon epithelial cell line appears to have been completely TIFA dependent and did not rely on NOD1 activation or sensing of the NOD1- and TIFA-independent bacterially derived factor.

### Gastric epithelial cells robustly respond to bacterially derived HBP.

To assess whether AGS cells are able to produce IL-8 in response to HBP stimulation, we reversibly permeabilized control or *TIFA* KO AGS cells with digitonin and treated them with *Neisseria* lysates containing HBP (Δ*gmhB*) or heptose-7-monophosphate (HMP; Δ*hldA*) (26). Control AGS cells treated with HBP showed an accumulation of IL-8 over a 24-h time period, whereas HMP-treated cells showed substantially lower IL-8 responses. Notably, when we assessed *TIFA* KO AGS cells treated with HBP, we found the same reduction in IL-8 responses as in control targeted AGS cells treated with HMP (Fig. 2D; Fig. S1D). These data suggest that AGS cells recognize HBP and activate TIFA to drive the NF-κB-dependent IL-8 response.

Published reports have demonstrated that HBP-inducible phosphorylation of TIFA at the T9 amino acid drives intermolecular oligomerization, which recruits and activates its downstream signaling partner TRAF6 (25, 27, 28). Therefore, we assessed whether the T9 residue is essential for TIFA activation in AGS cells during *H. pylori* infection. We stably complemented *TIFA* KO cells with either wild-type N-terminally tagged TIFA or a T9A substitution containing TIFA. We found that, similarly to *TIFA* KO AGS cells, IL-8 responses were reduced in the T9A complemented cells. *H. pylori* infection of both N- and C-terminally tagged wild-type complemented cells resulted in control levels of IL-8 (Fig. 2E; Fig. S1E). These data are consistent with previous studies and suggest that in AGS cells, *H. pylori*-induced inflammation requires phosphorylation of TIFA at T9 in order to activate TRAF6 and propagate the NF-κB-driven immune response.

To determine whether *H. pylori* cell lysates containing HBP could activate NF-κB in a TIFA-dependent manner, we treated NF-κB–luciferase reporter human embryonic kidney (293T) cells with various ligands and measured luciferase reporter activity. 293T cells have high transfection efficiency and lack endogenous TLR and some nucleic acid-sensing pathways (32, 33), allowing dissection of the TIFA-dependent response with little contribution of other PAMP sensing pathways. Consistent with published data (25, 28), we found that wild-type reporter cells robustly responded to HBP, but not to the upstream monophosphorylated derivative HMP, and that the induction of NF-κB–luciferase activity was largely dependent on TIFA. When we treated the reporter cells with G27 *H. pylori* cell lysates (Fig. 2F; Fig. S1F) or lysates from different *H. pylori* strain backgrounds (Fig. S2B), only the wild-type 293T cells responded and not the *TIFA* KO cells, showing a greater than 100-fold induction of NF-κB–luciferase. Both wild-type and *TIFA* KO cells responded to a TIFA-independent stimulus, tumor necrosis factor alpha (TNF-α) (Fig. 2F; Fig. S1F). Together, these data confirm that *cag*-T4SS-dependent delivery of *H. pylori* HBP into host epithelial cells triggers the activation of TIFA, leading to NF-κB activation and subsequent production of IL-8.

### *H. pylori* heptose biosynthesis mutants are filamentous and have delayed growth.

**FIG 3:**
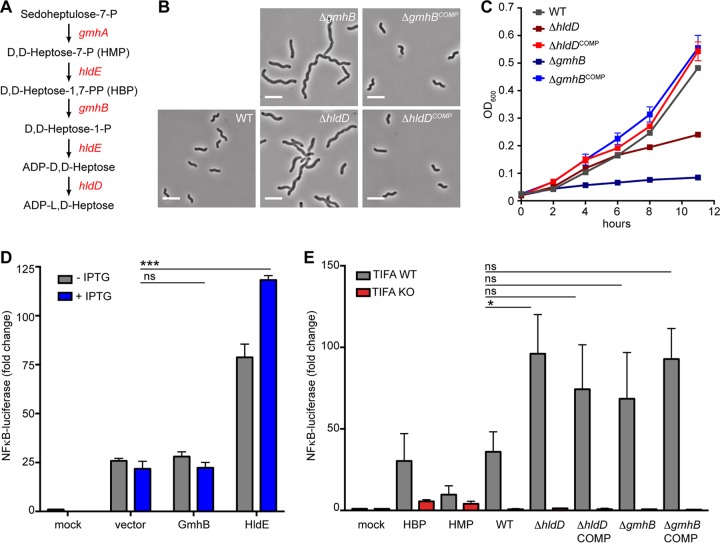
*H. pylori* ADP*-*heptose synthesis mutants are filamentous and have pronounced growth defects. (A) Predicted ADP-heptose biosynthesis pathway in *H. pylori*. (B) Phase-contrast images of wild-type cells or *H. pylori* mutants lacking the indicated genes and their respective complemented strains. Scale bars, 5 μm. (C) Growth curve analysis of wild-type, mutant, and complemented strains as measured by OD_600_ over 12 h. Each strain was grown in triplicate, and data are represented as means with standard deviations. Data are representative of results from two independent experiments. (D) NF-κB luciferase activity in 293T cells treated with *E. coli* lysates from cells expressing the indicated *H. pylori* enzymes with or without the addition of 1 mM isopropyl-β-d-thiogalactopyranoside (IPTG). (E) NF-κB luciferase activity in wild-type or *TIFA*-targeted 293T cells treated with *N. meningitides*-derived HBP (Δ*gmhB*), HMP (Δ*hldA*), or *H. pylori* cell lysates from strains of the indicated genotype. Data in panels D and E were normalized to signal from cotransfected *Renilla* luciferase plasmid and are represented as normalized fold changes from mock-treated samples. Each condition was tested in triplicate, with bars showing means with standard deviations, and data are representative of results from at least two independent experiments. Statistical significance was determined using ANOVA with Bonferroni correction for multiple comparisons. ns, not significant (*P* > 0.05); *, *P* < 0.05; ***, *P* < 0.001.

In Gram-negative bacteria, the biosynthesis of d,d-heptose and l,d-heptose, which are incorporated into the core oligosaccharide of LPS, occurs in the bacterial cytosol by the activity of highly conserved enzymes. In *E. coli*, the reaction begins with the conversion of sedoheptulose-7-P to d,d-heptose-7-P (HMP) by isomerase GmhA, followed by the heptokinase activity of bifunctional HldE to generate d,d-heptose-1,7-PP (HBP, the molecule that activates TIFA). HBP is then converted to d,d-heptose-1-P by phosphatase GmhB, which is further modified by the ADP-transferase activity of HldE to form ADP-d,d-heptose. ADP-d,d-heptose is then converted to ADP-l,d-heptose by epimerase HldD. WaaC then transfers the first l,d-heptose to 3-deoxy-d-manno-octulosonic acid (Kdo) for ongoing lipopolysaccharide synthesis (34). Sequenced *H. pylori* strains contain conserved homologues of the four *E. coli* enzymes involved in ADP-heptose biosynthesis (Fig. 3A). Using a phosphate release assay, Yu et al. showed that when recombinant *H. pylori* enzymes GmhA, HldE, and GmhB were combined with a sedoheptulose-7-P substrate, significantly more phosphate was released than was seen with the enzymes alone or with an enzyme mixture that was missing HldE, supporting the hypothesis that these proteins function similarly to the ADP-heptose biosynthesis pathway described for other Gram-negative bacteria (35). Furthermore, the crystal structure of *H. pylori* HldD revealed that both the substrate binding site and the overall structure were highly similar to those of the HldD of *E. coli*, suggesting a conserved enzymatic function (36). It has also been shown that deletion of several enzymes involved in assembly of the highly conserved LPS core in *H. pylori* results in mutants that are unable to colonize mice, typifying the essential nature of most PAMPs (37, 38). Furthermore, despite the fact that *waaC* was the first LPS biosynthesis gene identified in *H. pylori*, attempts to generate deletion mutants proved unsuccessful (39).

In our study, using the *H. pylori* G27 strain, we attempted to generate deletion mutants for all four predicted heptose biosynthesis genes but were unable to delete *gmhA* or *hldE*. HPG27_814 is annotated as a hypothetical protein in the fully sequenced G27 genome (40). Using the Web-based Phyre2 protein structure prediction tool (41), we found that HPG27_814 showed 35% sequence identity and mapped with 100% confidence as a homologue of *E. coli* GmhB (data not shown). Deletion of *hldD* and the putative *gmhB* gene in G27 resulted in highly filamentous mutants that had a pronounced growth defect. Complementation of *hldD* and *gmhB* at the neutral *rdxA* locus restored the wild-type shape and growth rates (Fig. 3B and C). Our observations are consistent with a previous report showing that deletion of *hldD* in strain 26695 resulted in bacteria with truncated LPS (deep-rough phenotype), decreased growth rates, increased susceptibility to novobiocin, and decreased adherence to AGS cells (42). To our knowledge, analysis of *gmhB* mutants had not been undertaken in *H. pylori*. However, in our study, deletion of *gmhB* in strain G27 resulted in an even more pronounced growth defect than deletion of *hldD*. This suggests that, although the heptose biosynthesis enzymes are part of the same pathway, deletions of individual enzymes have differing effects on *H. pylori*. Furthermore, another recent study characterized a *gmhA* deletion mutant in strain 26695 (35). The results showed that, similarly to the *hldD* mutant, Δ*gmhA* bacteria displayed truncated LPS, decreased growth rates, and increased susceptibility to novobiocin and detergents and were less able to adhere to AGS cells. Our inability to generate a Δ*gmhA* mutant may reflect strain-specific differences between G27 and 26695. It is also possible that the Δ*gmhA* 26695 mutant generated by Yu et al. contains a suppressor mutation that partially alleviates the lethality of deleting *gmhA* and thus results in viable, albeit sickly, bacteria.

### *H. pylori* HldE drives HBP synthesis and TIFA-dependent NF-κB activation.

In order to determine whether *H. pylori* HBP is driving the TIFA-dependent NF-κB activation, we used an *E. coli* overexpression system where we placed *H. pylori hldE*, which encodes the enzyme that produces HBP, under the control of an IPTG (isopropyl-β-d-thiogalactopyranoside)-inducible promoter. We collected uninduced and IPTG-induced cell lysates and used them to treat NF-κB–luciferase reporter cells. We found that only the *E. coli* cells that expressed *H. pylori* HldE potently activated NF-κB, a response that was further enhanced by IPTG treatment. In contrast, *E. coli* expressing *H. pylori* GmhB, which converts HBP to HMP, showed background levels of NF-κB activation that were equivalent to those seen with bacteria expressing vector only in a representative experiment (Fig. 3D) and across three independent experiments (Fig. S3A). To confirm that the enhanced NF-κB activation by lysates from *H. pylori* HldE-expressing *E. coli* was TIFA dependent, we treated wild-type or *TIFA* KO reporter cells with a dilution series of lysates from IPTG-induced GmhB- or HldE-expressing bacteria. We standardized our input based on total protein levels measured by a bicinchoninic acid (BCA) protein assay and found that across the dilution series, lysates from HldE-expressing *E. coli* consistently showed increased NF-κB activation relative to GmhB-expressing bacteria and that the NF-κB signal was completely TIFA dependent (Fig. S3B). Since the ADP-heptose biosynthesis enzymes appear to function in *H. pylori* similarly to the manner in which they function in other Gram-negative bacteria and since HldE, the enzyme that synthesizes HBP, appears essential, we focused our analysis on enzymes downstream of HBP. We hypothesized that targeting *gmhB* might result in HBP accumulation within the bacteria and, thereby, in increased TIFA-dependent NF-κB activation. Using the NF-κB–luciferase reporter cells, we treated wild-type or *TIFA* KO cells with lysates generated from wild-type, Δ*gmhB*, or Δ*hldD* (encoding an enzyme downstream of GmhB) bacteria. We normalized our input based on the total protein estimation using a BCA protein assay, since optical density (OD) is not an accurate reflection of total cell number in working with filamentous mutants. We found that lysates from Δ*hldD* mutants induced statistically significantly more NF-κB (*P* = 0.05) than those from wild-type *H. pylori* (Fig. 3E). However, assessing data across three independently conducted experiments, we found no significant differences among the levels of NF-κB activation induced by the wild-type, Δ*gmhB*, and Δ*hldD* strain lysates and the levels induced by their respective complemented strain lysates (Fig. S3C). It is possible that *H. pylori* controls metabolic pathways to prevent excess cytosolic HBP accumulation in the absence of GmhB. Nevertheless, our overexpression data determined with *E. coli* suggest that *H. pylori* HldE generates HBP, which signals through TIFA, leading to NF-κB activation.

10.1128/mBio.01168-17.3 FIG S3  *H. pylori* HldE drives TIFA-dependent NF-κB activation. (A) NF-κB luciferase activity in 293T cells treated with *E. coli* lysates from cells expressing the indicated *H. pylori* enzymes with or without the addition of 1 mM isopropyl-β-d-thiogalactopyranoside (IPTG). Data were combined from the results from three independent experiments and are represented as means and standard deviations of fold changes relative to NF-κB activity in 293T cells treated with *E. coli* lysates expressing vector only without the addition of IPTG. (B) NF-κB luciferase activity in wild-type or *TIFA*-targeted 293T cells treated with the indicated dilutions of wild-type *H. pylori* or *E. coli* lysates from cells expressing *H. pylori* GmhB or HldE and treated with IPTG. Cell lysate input was normalized based on total protein estimated using a BCA protein assay. (C) NF-κB luciferase activity in wild-type or *TIFA*-targeted 293T cells treated with *H. pylori* cell lysates from strains of the indicated genotype. Data were combined from three independent experiments and are represented as means and standard deviations of fold changes relative to NF-κB activity in wild-type 293T cells stimulated with wild-type *H. pylori* lysate. Statistical significance was determined using ANOVA with Bonferroni correction for multiple comparisons. ns, not significant (*P* > 0.05); **, *P* < 0.01. Download FIG S3, TIF file, 0.4 MB. Copyright © 2017 Gall et al. 2017 Gall et al. This content is distributed under the terms of the Creative Commons Attribution 4.0 International license.

### CagA contributes to the late NF-κB-dependent IL-8 response in gastric epithelial cells.

**FIG 4:**
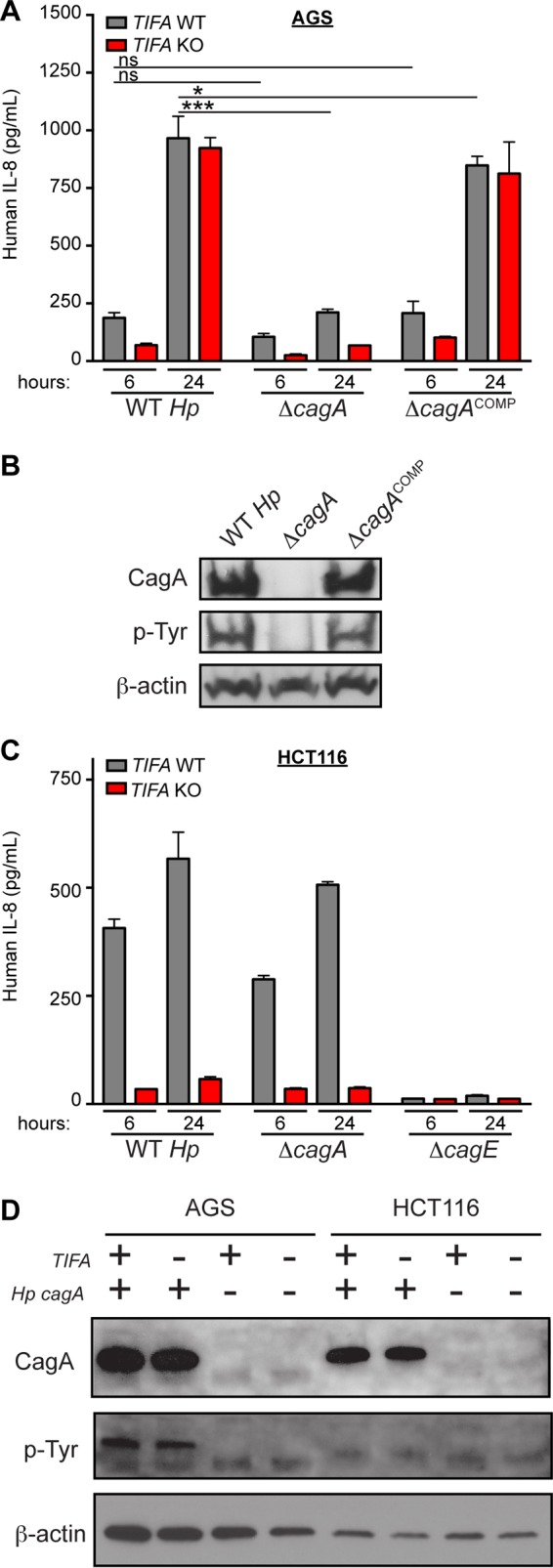
CagA contributes to the late NF-κB-driven immune response in gastric epithelial cells. (A) Control or *TIFA*-targeted AGS cells were cocultured with wild-type cells or with Δ*cagA* or Δ*cagA* complemented isogenic *H. pylori* mutants at MOI = 10, and IL-8 concentrations in the supernatant were measured by ELISA at the indicated time points. (B) Western blot analysis of cell lysates from control or *TIFA*-targeted AGS cells collected 24 h after coculture (MOI = 10) with wild-type cells or Δ*cagA* or Δ*cagA* complemented isogenic mutants. (C) Control or *TIFA*-targeted HCT116 cells were cocultured with wild-type cells or Δ*cagA* or Δ*cagE* isogenic mutants at MOI = 10, and IL-8 concentrations in the supernatant were measured by ELISA at the indicated time points. For the experiments shown in panels A and C, each condition was tested in triplicate, with bars showing means and standard deviations. Data are representative of results from at least 2 independent experiments. Statistical significance was determined using ANOVA with Bonferroni correction for multiple comparisons. ns, not significant (*P* > 0.05); *, *P* < 0.05; ***, *P* < 0.001. (D) Western blot analysis of cell lysates from control or *TIFA*-targeted AGS or HCT116 cells collected 24 h after coculture (MOI = 10) with wild-type cells or Δ*cagA* isogenic *H. pylori* mutants. In the Western blots shown in panels B and D, the phosphotyrosine (p-Tyr) blot indicates a band consistent with the size of CagA (∼130 kDa) and represents CagA that was translocated and phosphorylated inside the host cell.

Assessing *NOD1 TIFA* DKO AGS cells for IL-8 production in response to *H. pylori* infection, we found that IL-8 levels were substantially reduced at the earlier time points. However, in the absence of both NOD1 and TIFA signalling, we found that residual IL-8 accumulated over time, being especially evident at the 24-h time point (Fig. 2A). We hypothesized that CagA may have been driving this residual IL-8 signal since the N-terminal domain (amino acids 24 to 221) of CagA has been proposed to activate NF-κB (17, 43, 44). Indeed, when we cocultured wild-type or *TIFA* KO AGS cells with the *H. pylori* Δ*cagA* mutant, we found a significant decrease in the IL-8 response at the 24-h time point (Fig. 4A; Fig. S4), which was restored to wild-type levels when cells were cocultured with Δ*cagA* complemented *H. pylori* (Fig. S4). The reduced IL-8 levels were particularly evident in *TIFA* KO AGS cells, confirming that both TIFA activation and CagA detection contribute to the IL-8 response in AGS cells. We also showed that CagA is present and becomes phosphorylated within AGS cells cocultured with wild-type or Δ*cagA* complemented *H. pylori* (Fig. 4B). Examining HCT116 colon epithelial cells, where the IL-8 response to *H. pylori* appears to be almost entirely TIFA dependent, we did not observe a significant difference in IL-8 levels in cells treated with wild-type or isogenic *ΔcagA* mutant strains (Fig. 4C). We also observed that, while in AGS cells wild-type *H. pylori* delivered CagA into the host cell, where it was phosphorylated by host kinases, in HCT116 cells CagA was not phosphorylated, suggesting either that CagA is not delivered into these cells or that HCT116 cells are unable to phosphorylate translocated CagA (Fig. 4D).

10.1128/mBio.01168-17.4 FIG S4  CagA contributes to the late NF-κB-driven immune response in gastric epithelial cells. Control or *TIFA*-targeted AGS cells were cocultured with wild-type, Δ*cagA*, or Δ*cagA*-complemented isogenic *H. pylori* mutants at MOI = 10, and IL-8 concentrations in the supernatant measured by ELISA at the indicated time points. Data from two independent experiments were combined and are represented as means and standard deviations of fold changes in IL-8 concentrations relative to control targeted AGS cells cocultured with wild-type *H. pylori* for 6 h. Statistical significance was determined using ANOVA with Bonferroni correction for multiple comparisons. ns, not significant (*P* > 0.05); ***, *P* < 0.001. Download FIG S4, TIF file, 0.1 MB. Copyright © 2017 Gall et al. 2017 Gall et al. This content is distributed under the terms of the Creative Commons Attribution 4.0 International license.

## DISCUSSION

**FIG 5:**
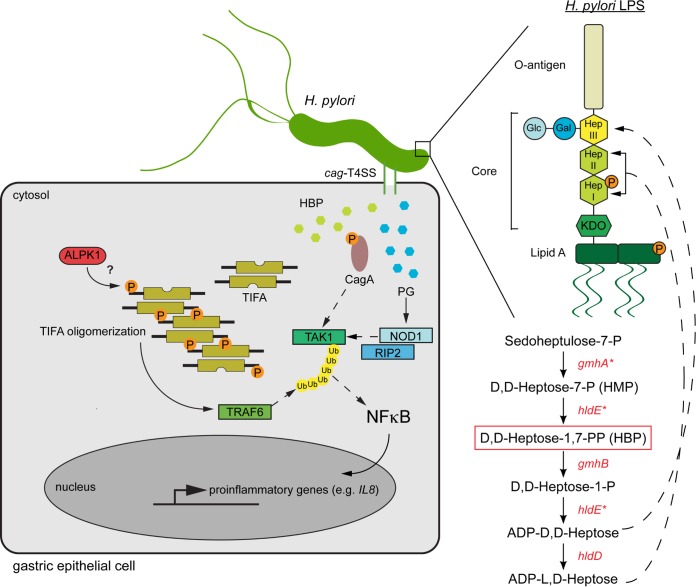
Model of *H. pylori cag-*T4SS-dependent, NF-κB-driven innate immune response in gastric epithelial cells. A schematic representation of *H. pylori* factors that are delivered through the *cag*-T4SS into gastric epithelial cells to initiate the NF-κB-mediated inflammatory response is shown. The response is initiated by cytosolic delivery of HBP that activates TIFA, which may be phosphorylated at the Thr9 residue by kinase ALPK1 (27). Phosphorylated TIFA forms oligomers that act as a scaffold to recruit and activate TRAF6, which in turn activates transcription factor NF-κB, which translocates into the nucleus and upregulates a suite of proinflammatory genes, including the IL-8 gene. Following TIFA activation, peptidoglycan (PG) delivered through the *cag*-T4SS activates NOD1, which ultimately amplifies NF-κB activation. Finally, CagA associates with host TAK1 and enhances its polyubiquitination, which is mediated by the E3 ligase activity of TRAF6. Polyubiquitinated TAK1 then activates NF-κB, further amplifying the proinflammatory cascade (43, 49). The HBP that is delivered through the *cag*-T4SS to activate TIFA is generated through the ADP-heptose biosynthesis pathway in the bacterial cytosol. Four *H. pylori* enzymes are involved in converting sedoheptulose-7-P into ADP-l,d-heptose. Two of these enzymes (marked with an asterisk), GmhA and HldE, appear to be essential in the G27 *H. pylori* strain. ADP-l,d-heptose and ADP-d,d-heptose are incorporated into the highly conserved core structure of *H. pylori* LPS, which consists of a hexa-saccharide (glucose—galactose-d,d-heptose [Hep III]—l,d-heptose [Hep II]—l,d-heptose [Hep I]—KDO). The variable O-antigen is directly attached to the Hep III molecule (37). The LPS molecule is assembled in the bacterial cytosol, transported across the periplasm, and flipped across the outer bacterial membrane.

Using CRISPR/Cas9 gene targeting of specific pathogen recognition pathways, we revealed that TIFA is involved in the human cellular response to *H. pylori*, and we were able to dissect the relative contributions of the *H. pylori*-derived factors to the NF-κB-mediated immune response in gastric epithelial cells. While it was previously accepted that NOD1 activation following *H. pylori* cell wall fragment (PG) detection is the main proinflammatory signal that leads to IL-8 production, here we show that *H. pylori* HBP-dependent TIFA activation also plays a critical role in the early immune response to *H. pylori* (Fig. 5). Furthermore, we reveal a previously unrecognized mechanism by which HBP gains access to the cytosolic TIFA pathway, since *H. pylori* HBP is delivered directly to the host cell cytosol through the *cag*-T4SS. It remains an open question whether HBP delivery to the host is an inadvertent consequence of forming the *cag*-T4SS that connects the bacterial and host cytosol or whether *H. pylori* HBP delivery to the host instigates an inflammatory response that benefits the bacteria, such as by the recruitment of regulatory T cells and activation of other tolerogenic mechanisms that prevent bacterial clearance and support chronic colonization (14, 45).

It was recently demonstrated on the basis of the rates of cytosolic replication by invasive bacteria that TIFA activation fine-tunes the downstream inflammatory response (28). That study suggested that TIFA signaling is one mechanism by which intestinal epithelial cells can potentially distinguish commensal bacteria from pathogenic invaders. Interestingly, the temporal pattern of NOD1 and TIFA activation in the context of invasive *Shigella* infection is the inverse of what we observed during *H. pylori* infection: the NOD1 response constitutes the initial response to *Shigella*, and TIFA is activated later (28), while the HBP response occurs earlier with *H. pylori*. This difference may be attributable to the very different lifestyles of these bacteria. While HBP slowly accumulates within the mammalian cell as *Shigella* grows, a bolus of the cytosolic HBP present within *H. pylori* can presumably be rapidly delivered as the *cag*-T4SS penetrates the host cell, rapidly activating the TIFA pathway.

The impact of the CagA toxin on *H. pylori*-induced inflammation is somewhat controversial. Because Δ*cagA H. pylori* strains induce a robust inflammatory response, a number of studies concluded that CagA is dispensable for the *cag*-T4SS-dependent NF-κB-driven immune response (46–48). However, a consensus has recently emerged supporting the idea that CagA itself can activate the NF-κB inflammatory pathway since transfection with CagA expression constructs leads to NF-κB activation (17) and that CagA physically binds the immune signaling component TAK1 to promote NF-κB activation (43, 49). Our data support the latter studies, showing that CagA can drive an NF-κB-mediated immune response. However, we found that CagA likely activates this pathway later than TIFA and NOD1. Because the TIFA and NOD1 pathways converge on TRAF6 activation, it is possible that initial activation of these pathways potentiates CagA interaction with TAK1 (Fig. 5) and that CagA then further amplifies and/or sustains NF-κB activation. Further work is needed to fully elucidate the mechanism by which CagA activates NF-κB.

Several groups have recently provided insight into the LPS structure of various commonly studied *H. pylori* strains (37, 50–53). Li et al. proposed a redefinition of the *H. pylori* LPS structure to contain lipid A, a short, highly conserved core hexa-saccharide domain composed of glucose-galactose-d,d-heptose-l,d-heptose-l,d-heptose-2-KDO (keto-3-deoxyoctulosonic acid) and the direct attachment of the variable O-antigen to the d,d-heptose molecule (37). This is in contrast to the LPS structures of other Gram-negative bacteria, which contain an inner core resembling the hexa-saccharide domain of *H. pylori* and an outer core to which the O-antigen is attached. Studies have shown that deletion of enzymes involved in the assembly of the highly conserved LPS core in *H. pylori* results in bacteria that are unable to colonize mice (14, 37, 38, 45). Our own data suggest that *hldE* and *gmhA*, at least in the G27 strain background, appear to be essential in *H. pylori* and that deletion of *hldD* and *gmhB* results in highly filamentous and slow-growing bacteria. The enzymes involved in the biosynthesis of ADP-heptose and assembly of the core LPS oligosaccharide present bacterial pathways that can potentially be therapeutically targeted.

Our data suggest that TIFA, NOD1, and CagA all contribute to the NF-κB-driven inflammatory response in gastric epithelial cells; however, how much each of these pathways contributes to natural infection remains an open question. Although AGS cells represent a useful and tractable model system to study *H. pylori* interaction with gastric epithelial cells, they do not fully recapitulate the complexity of primary gastric epithelial cells. Interestingly, gene expression analysis of primary human stomach tissue validates the idea that *TIFA* and *NOD1* are both expressed, supporting their physiological relevance to *H. pylori* infection observed in our study. Furthermore, primary stomach tissue has much higher expression levels of *TIFA* than of *NOD1*, which may reflect the relative contributions of the two pathways to *H. pylori* detection (28). Future studies will focus on assessing NOD1 and TIFA innate immune signaling pathways in primary gastric organoids and animal models of infection to further elucidate their respective contributions to *H. pylori* detection.

How *H. pylori* is able to maintain a chronic infection despite a robust host immune response has remained a mystery. To address that question, we must first understand the underlying host inflammatory pathways that *H. pylori* activates and subverts. Our study revealed a previously unappreciated role of TIFA in the innate immune response initiated in *H. pylori*-infected gastric epithelial cells. Furthermore, we demonstrate, for the first time, that HBP can be directly delivered to host cells through a bacterial secretion system where it potently activates TIFA and drives the downstream NF-κB-mediated inflammatory response. Gaining a comprehensive view of the early signaling events that take place at the interface of *H. pylori* and gastric epithelial cells is ultimately required to determine how this host-pathogen interaction can be manipulated in the host’s favor. Our report adds a critical piece to our understanding of the host immune response to an important human pathogen.

## MATERIALS AND METHODS

### CRISPR/Cas9 gene targeting and complementation.

CRISPR/Cas9 gene targeting of AGS cells was performed as described by Gray et al. (54). Briefly, we used a single lentiviral plasmid that expresses the gene targeting guide RNA under the control of a U6 promoter, as well as an MND promoter-driven Cas9-T2A-puromycin or -blasticidin resistance cassette (pRRL lentiviral vector). 293T cells were transfected with 1.5 μg pVSV-G, 3 μg psPAX-2, and 6 μg pRRL lentiviral vector. Lentiviral supernatants were collected 48 h later and used to transduce AGS cells. AGS cells were then selected with puromycin (0.5 μg/ml) or blasticidin (12.5 μg/ml) for 7 to 10 days. As a control, cells were transduced with pRRL expressing Cas9 only or a human nontargeting control guide RNA, as described by Sanjana et al. (55). *NOD1*-targeted cells were clonally selected, and the gene targeting was verified by TOPO cloning and Sanger sequencing. *TIFA* targeting was verified using an *in vitro* RNA-guided engineered nuclease–restriction fragment length polymorphism (RGEN-RFLP) assay, as previously described (56). The following guide RNA sequences were used: for *NOD1*, 5′-GTTTTCAGTAATTGAATGTGG-3′; for *TIFA*, 5′-CAGATGACGGTTTACCATCC-3′. To restore TIFA in targeted cells, the TIFA coding DNA sequence (CDS) was cloned into the pRRL lentiviral backbone to generate an MND promoter-driven TIFA-T2A-zeocin plasmid. *TIFA*-targeted cells were subsequently transduced with pRRL-MND-TIFA-T2A-zeocin plasmid, as described above, and selected with 250 μg/ml zeocin for 10 to 14 days. To ensure that the complementation construct was CRISPR resistant, we introduced single nucleotide polymorphisms (SNPs) into the guide RNA-targeted genomic sequence that resulted in synonymous mutations, which maintain the TIFA amino acid sequence but prevent guide RNA binding and Cas9 cutting. The following single oligonucleotide was used in a site-directed mutagenesis reaction to alter the TIFA CDS: 5′-GAGACAGTAACTTGTCTCCAaATGACaGTcTAtCAcCCaGGCCAGTTGCAGTGTGGAATA-3′ (the guide RNA sequence is underlined, and the introduced SNPs are in lowercase). In addition, C-terminal 6×His and V5 tags and N-terminal FLAG tag were added, as indicated, to the TIFA CDS to allow downstream protein expression analysis.

### Bacterial strains.

G27, a clinical isolate of *H. pylori* that contains the *cag* pathogenicity island (57), or isogenic mutants were grown at 37°C in a trigas incubator equilibrated with 10% oxygen, 10% carbon dioxide, and 80% nitrogen on solid media containing horse blood agar or in liquid culture containing brucella broth (BD Biosciences) with 10% heat-inactivated fetal bovine serum (FBS) (Gemini-Benchmark), as previously described (58). Isogenic knockout mutants were constructed using a vector-free allelic replacement strategy where >60% of the CDS was replaced by a chloramphenicol resistance cassette and selected with 15 μg/ml chloramphenicol, as previously described (59). To generate complementation mutants, we integrated a wild-type copy of the deleted gene at a neutral locus: either *rdxA* with metronidazole selection (60) or an intragenic region between HPG27_186 and HPG27_187 (McGee locus) with kanamycin selection (61). The primers used for generating *H. pylori* mutants are listed in Table S1 in the supplemental material. *E. coli* DH5α, HST08, or BL21 cells were grown and transformed according to standard methods (62). For induced protein overexpression experiments, *H. pylori gmhB* and *hldE* genes were amplified and cloned into pET15b vector (Novagen). *E. coli* BL21 cells were transformed, selected with 100 μg/ml ampicillin, and grown to an optical density at 600 (OD_600_) of 0.6. Cells were then induced with 1 mM IPTG for 4 h and harvested by centrifugation. Cell pellets were then processed to obtain cell lysates as described above for NF-κB–luciferase assays or were resuspended in Laemmli buffer with β-mercaptoethanol for protein expression analysis by Western blotting.

10.1128/mBio.01168-17.5 TABLE S1  Primers for mutant *H. pylori* design. Primer sequences homologous to sequences of the antibiotic resistance cassette or integration locus are represented by lowercase lettering. Abbreviations: CM, chloramphenicol; Mtz, metronidazole; Kan, kanamycin. Download TABLE S1, PDF file, 0.1 MB. Copyright © 2017 Gall et al. 2017 Gall et al. This content is distributed under the terms of the Creative Commons Attribution 4.0 International license.

### Cell treatments and analysis.

AGS cells, from a human gastric adenocarcinoma cell line (ATCC CRL-1739), were grown in Dulbecco’s modified Eagle’s medium (DMEM) (Gibco) supplemented with 10% heat-inactivated FBS (Gemini-Benchmark). HCT116 cells, from a human colon adenocarcinoma cell line (ATCC CCL-247), were grown in McCoy’s 5A media (ATCC) supplemented with 10% FBS. *TIFA* KO HCT116 cells were generated using CRISPR/Cas9 gene targeting, as previously described (28). For coculture with *H. pylori*, AGS or HCT116 cells were seeded at 1 × 10^5^ cells/well in 24-well plates 16 h prior to infection. The day of infection, medium was removed from human cells and mid-log-phase *H. pylori* was added at multiplicity of infection of 10:1 resuspended in either DMEM–10% FBS–20% brucella broth for the AGS cells or McCoy’s 5A–10% FBS–20% brucella broth for the HCT116 cells. Supernatants from three individual wells per experimental condition were collected at the indicated time points and assayed for the IL-8 concentration using a human IL-8 enzyme-linked immunosorbent assay (ELISA) kit according to the instructions of the manufacturer (BioLegend). As indicated, cells were also treated with C12-iE-DAP (InvivoGen), IL-1β (BioLegend), or TNF-α (PeproTech). NF-κB–luciferase assays were performed as previously described (25) using a Dual-Glo luciferase assay system (Promega). Wild-type or *TIFA* KO human embryonic kidney (HEK) 293T cells were plated in triplicate and cotransfected with 90 ng NF-κB–luciferase and 10 ng *Renilla* luciferase plasmids and then treated the following day with *Neisseria* lysates containing HBP, HMP, or cell lysates from *H. pylori* or isogenic mutants in 5 μg/ml digitonin-containing permeabilization buffer (63). To obtain cell lysates, a volume of mid-log-phase *H. pylori* culture equivalent to an OD_600_ of 1 was pelleted by centrifugation, washed once with phosphate-buffered saline (PBS), resuspended in 100 μl of water, boiled for 10 min, and centrifuged to remove cell debris and the supernatant was passed through a 0.22-μm-pore-size syringe filter. Input for luciferase assays was normalized using a BCA protein assay kit (Pierce), and 1 μl of normalized lysate was used to stimulate 293T reporter cells.

### Immunoblotting.

After 24 h of coculture with *H. pylori*, AGS cells were washed with ice-cold PBS and lysed in Laemmli buffer with β-mercaptoethanol and boiled for 5 min. For CagA translocation and phosphorylation analysis, cell lysates were separated on Mini-Protean TGX polyacrylamide gels (Bio-Rad) and blotted for anti-phosphotyrosine (4G10; EMD Millipore) and beta-actin (Cell Signaling Technology, Inc.) overnight at 4°C. Membranes were then blocked for several hours in 5% nonfat milk–Tris-buffered saline with Tween 20 (TBST) and then incubated with anti-CagA antibody (Santa Cruz; sc-28368) overnight at 4°C.

### Statistical analysis.

The statistical significance of differences between groups was assessed using one-way analysis of variance (ANOVA) with Bonferroni correction for multiple comparisons. Statistical differences were assessed within individual experiments based on mean values from 3 independent samples per experimental condition, as well as across at least two independently performed experiments. *P* values greater than or equal to 0.05 were considered statistically significant. All analyses were performed using Prism v7.0 software (GraphPad).
