# Supplementary figures and images for: TIFA Signaling in Gastric Epithelial Cells Initiates the cag Type 4 Secretion System-Dependent Innate Immune Response to Helicobacter pylori Infection
Source: mBio. 2017 Aug 15;8(4):e01168-17. doi: 10.1128/mBio.01168-17 (PMC5559637; doi:10.1128/mBio.01168-17)

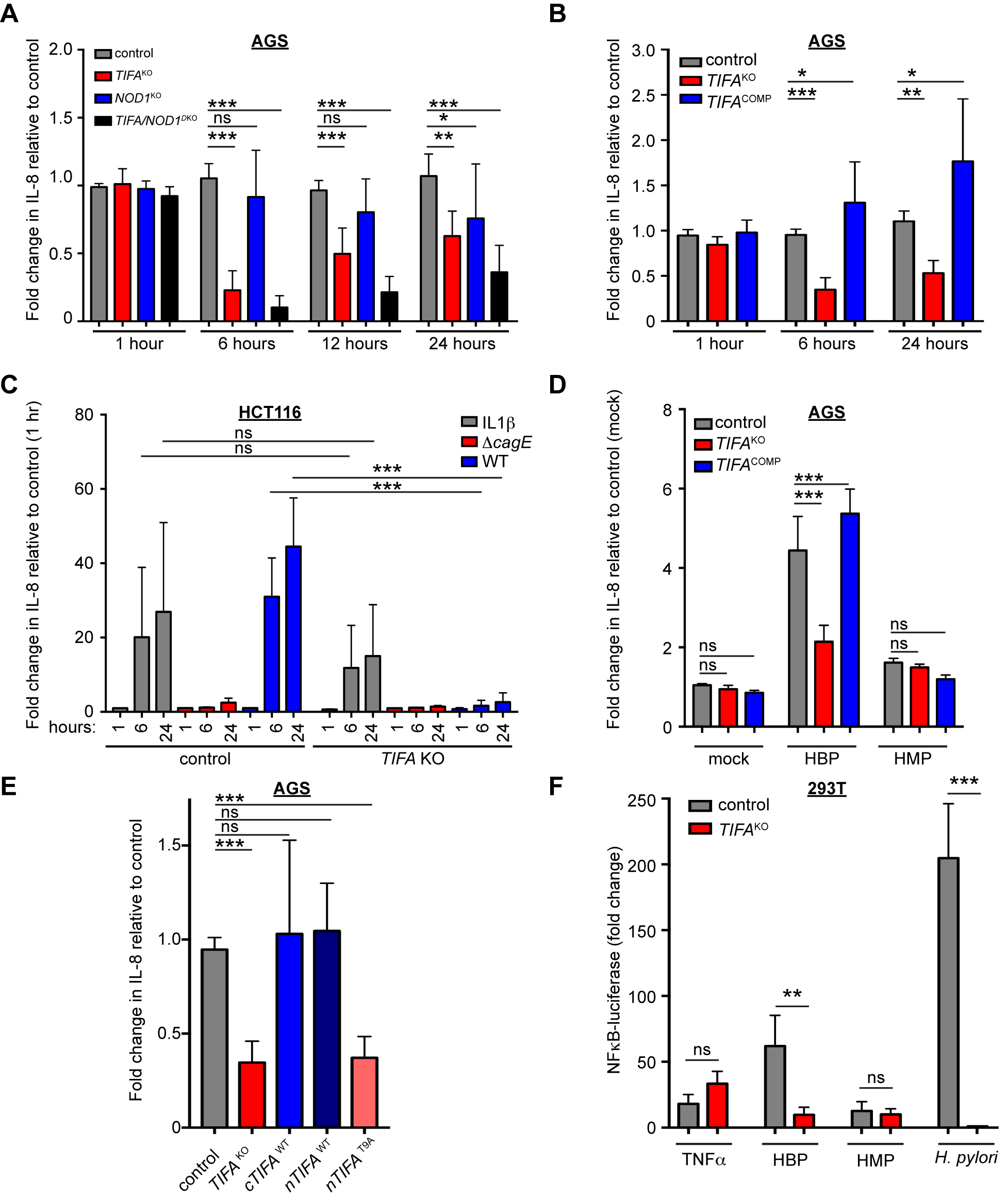

Supplement: FIG S1 [file mbo004173429sf1.tif]

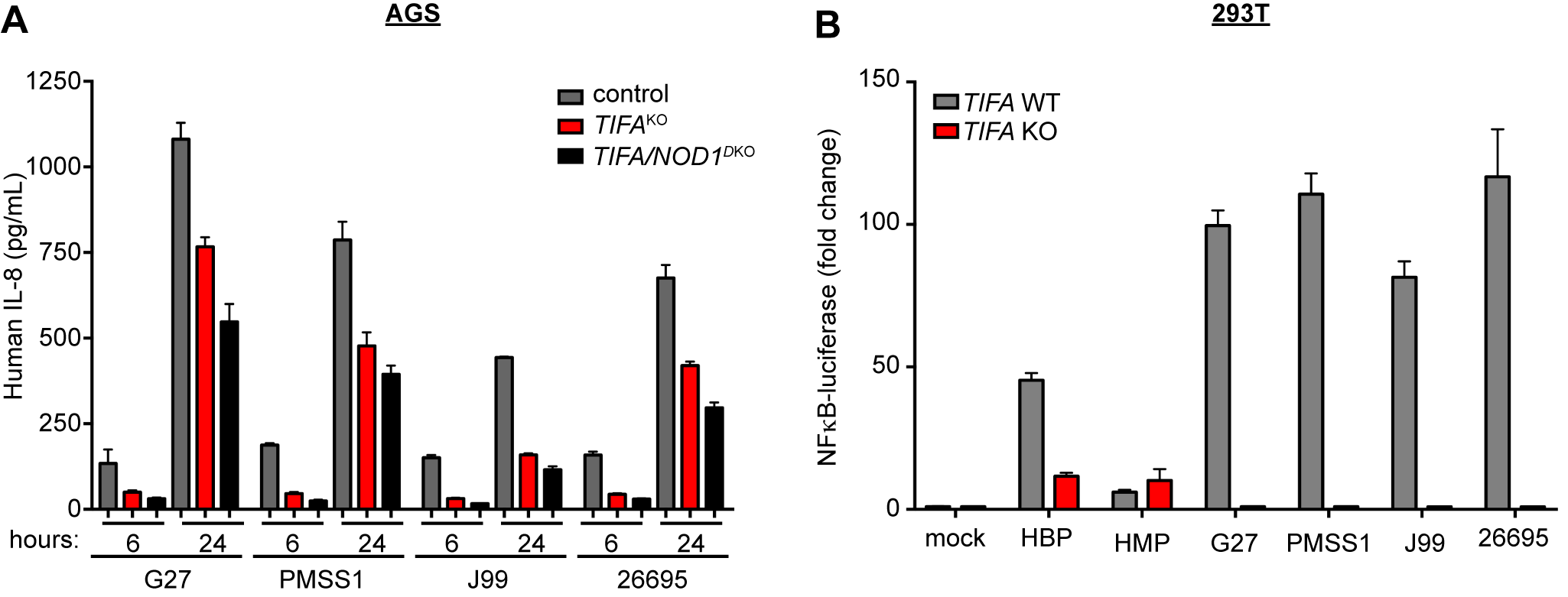

Supplement: FIG S2 [file mbo004173429sf2.tif]

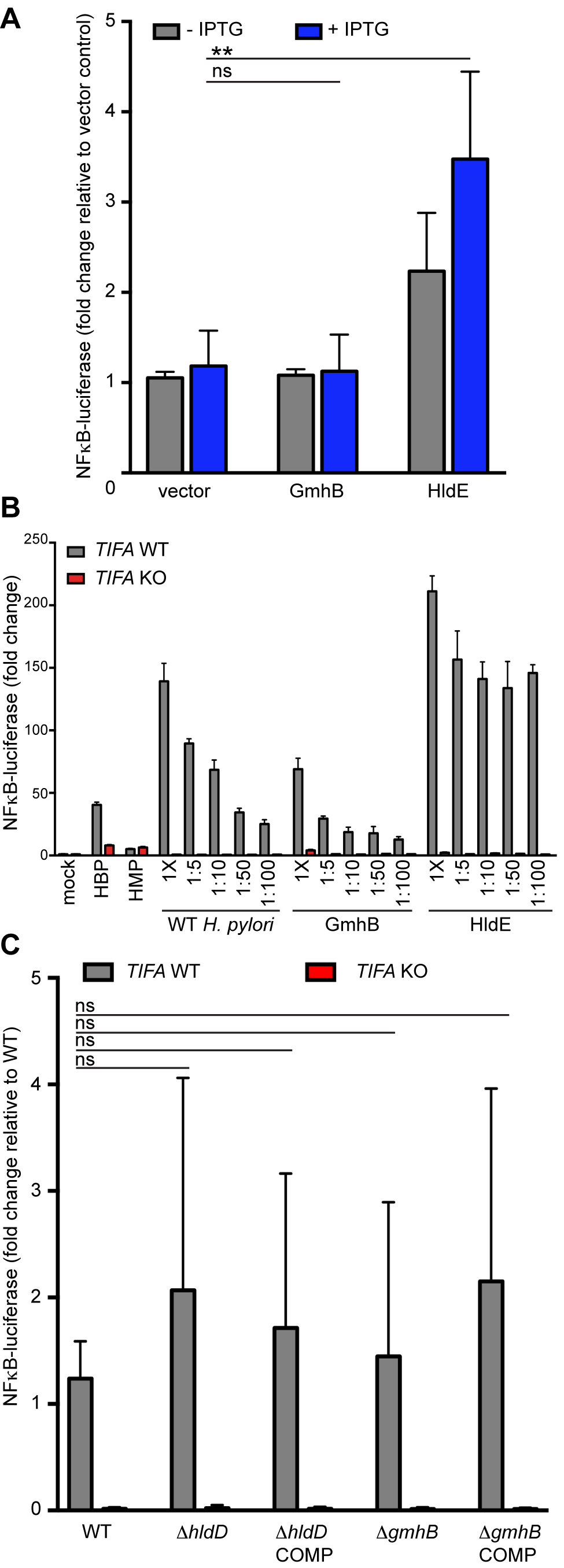

Supplement: FIG S3 [file mbo004173429sf3.tif]

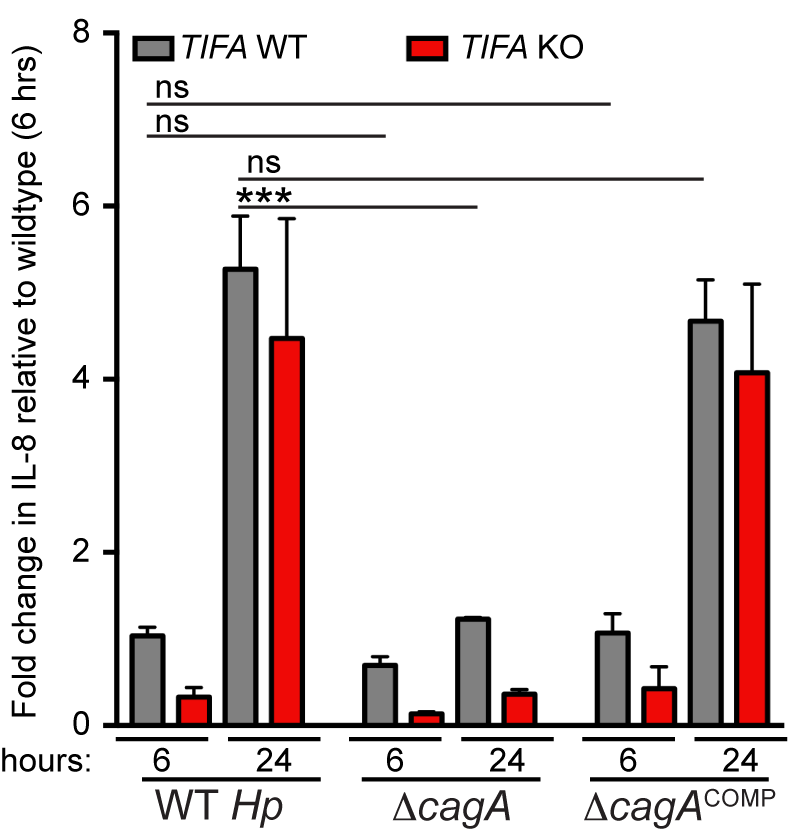

Supplement: FIG S4 [file mbo004173429sf4.tif]
