# Supplementary material for: TIFA Signaling in Gastric Epithelial Cells Initiates the cag Type 4 Secretion System-Dependent Innate Immune Response to Helicobacter pylori Infection
Source: mBio. 2017 Aug 15;8(4):e01168-17. doi: 10.1128/mBio.01168-17 (PMC5559637; doi:10.1128/mBio.01168-17)
Supplement: TABLE S1 [file mbo004173429st1.pdf]

Table S1: primers for mutant *H. pylori* design

Strain name	G27 gene number	Selection	Primer name	Primer sequence (5’ → 3’)
Δ*cagE*	503	CM	Δ*cagE* primer 1	CTTTCTGTCATTGCGCGGAG
			Δ*cagE* primer 2	GTTCAGTGATTTCGCCTTCCA
			Δ*cagE* primer 3	tccacttttcaatctatatccTATGATTAGCTTTTTTTGT
			Δ*cagE* primer 4	ccagtttctcgcactgataaACTGTATTTGTCAAAGAAAT
Δ*hldD*	813	CM	Δ*hldD* primer 1	GCGACATGTTAGCTGGCTTG
			Δ*hldD* primer 2	CGCTCCGCCTAAATTCTTGC
			Δ*hldD* primer 3	tccacttttcaatctatatccGATTAAATTCTTAAAATGC
			Δ*hldD* primer 4	ccagtttctcgcactgataaTAGCATTTTAAAAGAGCATT
Δ*gmhB*	814	CM	Δ*gmhB* primer 1	GGCCTTTCTTTTGGGCTTGG
			Δ*gmhB* primer 2	ATGCCCTAAAGAACTGGGGC
			Δ*gmhB* primer 3	tccacttttcaatctatatccTCGCATGGTTTAGTAATTCA
			Δ*gmhB* primer 4	ccagtttctcgcactgataaCATGTTAGCTGGCTTGAACG
Δ*cagA*	507	CM	Δ*cagA* primer 1	CTAGCCTTTAGACGCCTGCA
			Δ*cagA* primer 2	AGCGCGATCGATTTGCTCTA
			Δ*cagA* primer 3	tccacttttcaatctatatccATCAACTTTAAGAAAAGCCA
			Δ*cagA* primer 4	ccagtttctcgcactgataaCGAGTTGAACAATGCTGTAA
Δ*hldD* comp	813	Mtz	*rdxA*::*hldD* primer 1	agcgttaatggtggtatgctcCAATGCAAAGATCTTAAAG
			rdxA::*hldD* primer 2	CTTTAAGATCTTTGCATTGGAGCATACCACCATTAACGCT
			rdxA::*hldD* primer 3	AAAAATCCTAGTCGTAGGCGCATGGGCGTGAGCTTAATGG
			rdxA::*hldD* primer 4	ccattaagctcacgcccatgcGCCTACGACTAGGATTTTT
Δ*gmhB* comp	814	Mtz	*rdxA*::*gmhB* primer 1	agcgttaatggtggtatgctcGCATGATCATAAAGGAGCG
			rdxA::*gmhB* primer 2	CGCTCCTTTATGATCATGCGAGCATACCACCATTAACGCT
			rdxA::*gmhB* primer 3	GATAAGAATGCGTTATATTGCATGGGCGTGAGCTTAATGG
			rdxA::*gmhB* primer 4	ccattaagctcacgcccatgcAATATAACGCATTCTTATC
Δ*cagA* comp	507	Kan	McGee:*cagA* primer 1	tcgataccgtcgacctcgagcGCAACTCCATAGACCAC
			McGee:*cagA* primer 2	agagtaattctgtgggtaccTTAGCAAGGGGTGGT

*Note: primer sequence homologous to antibiotic resistance cassette or integration locus is represented by lowercase lettering
CM – chloramphenicol
Mtz – metronidazole
Kan – kanamycin
